# Laparoscopic Intervention to Pancreatic Pseudocyst Confers Short-Term Benefits: A Meta-Analysis

**DOI:** 10.1155/2021/7586338

**Published:** 2021-11-17

**Authors:** Yulin Guo, Shun Hu, Shuo Wang, Ang Li, Feng Cao, Fei Li

**Affiliations:** ^1^Department of General Surgery, Xuanwu Hospital, Capital Medical University, Beijing 100053, China; ^2^Research and Development Department, Sinovac Biotech Ltd, Beijing 100053, China

## Abstract

**Background:**

Surgical interventions for pancreatic pseudocyst (PP) are traditionally managed by an open surgical approach. With the development of minimally invasive surgical techniques, a laparoscopic surgical approach for PPs has been conducted increasingly with comparable outcomes. The present study was conducted to compare the efficacy and safety of surgical intervention for PPs between the laparoscopic approach and the open approach.

**Methods:**

Databases including Cochrane Library, PubMed, and EMBASE were searched to identify studies that compared the safety and efficacy of surgical intervention for PPs between the laparoscopic approach and the open approach (until Aug 1st 2020).

**Results:**

A total of 6 studies were eligible in qualitative synthesis. The laparoscopic approach was associated with less intraoperative blood loss (MD = −69.97; 95% CI: −95.14 to −44.70, *P* < 0.00001; *P*=0.86 for heterogeneity) and shorter operating time (MD = −33.12; 95% CI: −62.24 to −4.00, *P*=0.03; *P* < 0.00001 for heterogeneity). There was no significant difference found between the two approaches regarding the success rate and the recurrence rate. The postoperative complications and mortality rates were comparable between the two approaches.

**Conclusions:**

The laparoscopic approach for the surgical intervention of PPs is safe and efficacious with shorter-term benefits.

## 1. Introduction

Pancreatic pseudocyst (PP) is a localized collection of intrapancreatic and peripancreatic fluid surrounded by a well-defined wall of fibrous or granulation tissue, which results from acute pancreatitis, trauma, chronic pancreatitis, or pancreatic ductal obstruction [1, 2]. PP is not a true cyst which is sealed with an epithelial wall. PP contains fluid originated from pancreatic tissue due to the inflammation and pancreatic secretions leaked from the pancreatic ductal system.

PP accounts for about 80% of all pancreatic cysts. PP should initially be under conservative treatment because up to 85% could be spontaneously resolved within 4 to 6 weeks by its natural history. For those PPs that are symptomatic, larger than 6 cm, and/or last for more than 6 weeks, aggressive intervention should be taken to prevent infection, rupture, haemorrhage, and obstruction of the adjacent stomach, bowels, or bile ducts [3, 4].

Aggressive surgical interventions such as cystgastrostomy, cystduodenostomy, and Roux-en-Y cystjejunostomy could be performed for PPs according to their locations. Surgical interventions for PPs are traditionally managed by an open surgical approach with good long-term outcomes but noted complication rate [5]. As with the development and wide application of minimally invasive surgical techniques, the laparoscopic approach to PPs has been found to be associated with low morbidity and comparable outcomes compared to the standard open approach [6, 7]. However, there are few comparative studies that have compared the efficacy of the laparoscopic approach against the open approach until now. Also, this issue still needs to be well defined. The present study was conducted to compare the efficacy and safety of the surgical intervention for PPs between the laparoscopic approach and the open approach. The present study was organized and reported according to the PRISMA Checklist (Table S1).

## 2. Materials and Methods

### 2.1. Search Strategy for Studies

A comprehensive search was performed in PubMed, Cochrane Library, and EMBASE databases for eligible articles comparing laparoscopic surgical interventions with the open procedure for PPs regarding the safety and efficacy (until Aug 1, 2020). The search terms were as follows: (pancreatic or pancreas), pseudocyst, open, and (laparoscopic or laparoscopy). The comprehensive search was conducted with the combination of the abovementioned terms. While performing the study searching, referenced articles and related articles were manually and carefully screened to determine their potentiality.

### 2.2. Inclusion and Exclusion Criteria

The studies included should meet the following inclusion criteria: (1) Patients diagnosed with PPs. (2) Patients in one group had undergone the laparoscopic intervention for PPS, and patients in another group had undergone an open procedure. (3) For studies involving an overlapped population, the one with better quality was included. (4) The study should report no less than one of the outcomes of interest about the perioperative outcomes and mortality. The studies excluded involved the followings aspects: (1) Unpublished studies and studies presented as abstracts with unavailable full text. (2) Case series and case reports. (3) Letters, conferences, and reviews. We followed the methods of Guo et al. [8] in the process of study selection according to the inclusion and exclusion criteria.

Two authors evaluate the retrieved studies through reviewing the titles and abstracts to seek potential studies in an independent manner. Then, they carefully read the full texts of these studies referring to the abovementioned criteria. During this selection process, EndNote X6 software was used. Whenever there comes an inconsistency, a discussion would be held.

### 2.3. Data Extraction and Methodology Quality Assessment

We followed the methods of Guo et al. [8] in data extraction and methodology quality assessment. Data of interest were extracted by two authors in an independent manner. Data extracted contained the followings: name of the first author and year of publication, the number of patients, baseline characteristics, and study design. Perioperative results, postoperative morbidity, and mortality were the outcomes that we showed interest in.

The Newcastle–Ottawa Scale (NOS) was applied to examine the quality of cohort studies. Based on the NOS, the following items were scored: patient selection, comparability, and assessment of outcome. A study with a total score more than six was defined as moderate to high quality. Whenever there comes an inconsistency during this process, a discussion would be held.

### 2.4. Statistical Analysis and Calculation

All the data were statistically analyzed with Review Manager (Version 5.3, Cochrane Collaboration, Oxford, UK). Mean differences (MDs) with a 95% confidence interval (CI) were used for analyzing continuous data, while odds ratios (ORs) with a 95% CI were used for dichotomous data. When data were shown as median with range, the method reported by Hozo et al. [9] was adopted to calculate the mean and standard deviation. Heterogeneity across studies was assessed with *I*^2^.

## 3. Results

### 3.1. Search Results and Study Characteristics

**Figure 1:**
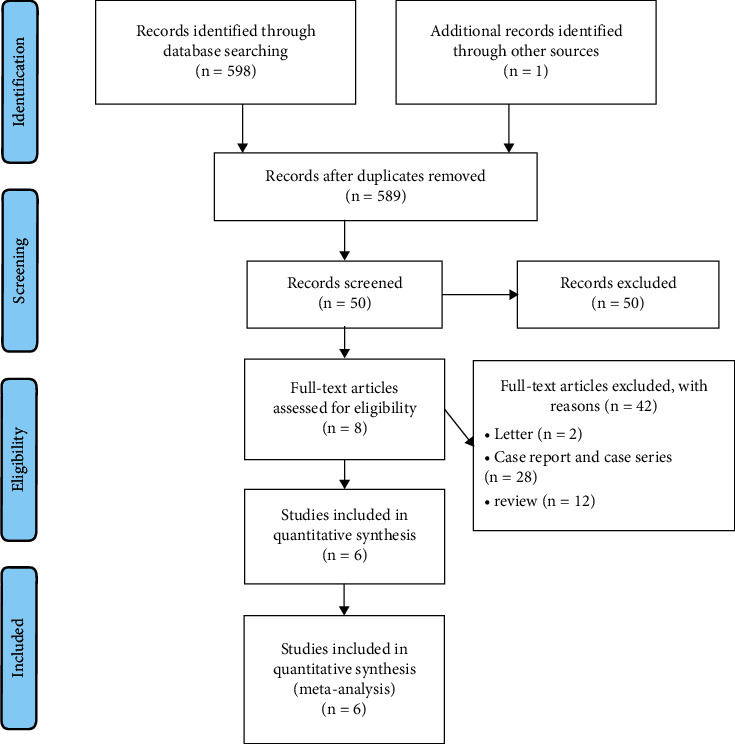
Flow diagram of the study.

A total of 599 studies were acquired after a systemic search. 10 duplicates were deleted. Then, through scanning titles and abstracts, 539 irrelevant studies, 2 letters, 28 case reports and case series, and 12 reviews were deleted. Among the remaining 8 studies, full text of every study was carefully read by referring to the inclusion and exclusion criteria. At last, 6 studies were eligible for qualitative synthesis (Table 1) [10–15]. The flow diagram of study selection is shown in Figure 1. A total of 513 patients were involved, with 318 patients in the laparoscopic group and 195 patients in the open group. Table 1 shows the basal characteristics of patients and general information of each included study. In the study conducted by Melman et al. [11], the sizes of pseudocysts and age of patients were different between the laparoscopic and the open groups. In the study conducted by Redwan et al. [14], the age of patients was also different between the two groups.

### 3.2. Quality Judgments of Studies

The Newcastle–Ottawa Scale (NOS) was used to evaluating the quality of included studies. For these cohort studies, one that acquires a score of more than six stars is considered as moderate to high quality. Table 1 shows all the included studies scored no less than six stars.

### 3.3. The Pooled Results

The intraoperative blood loss was reported by three studies, but the continuous data presented as median with range or the mean with standard deviation were only available from two of studies [12, 14]. Thus, regarding the two studies reporting the intraoperative blood loss, there were 64 patients involved. The intraoperative blood loss in the laparoscopic group was significantly lower than that of the open group (MD = −69.97; 95% CI: −95.14 to −44.70, *P* < 0.00001; *P*=0.86 for heterogeneity) (Table 2). Operating time was reported by five studies, with 64 patients in the laparoscopic group and 72 patients in the open group [10–14]. The operating time in the laparoscopic group was significantly shorter than that of the open group (MD = −33.12; 95% CI: −62.24 to −4.00, *P*=0.03; *P* < 0.00001 for heterogeneity) (Table 2).

The success rate for each surgical approach was reported by five studies, including 70 patients in the laparoscopic group and 88 patients in the open group [10–14]. There was no significant difference found between the two groups (OR = 1.25; 95% CI: 0.33 to 4.81, *P*=0.74; *P*=0.97 for heterogeneity) (Table 2). Data on recurrence rate was reported by four studies, with 60 patients in the laparoscopic group and 82 patients in the open group [11–14]. The recurrence rate of the laparoscopic group was comparable to that of the open group (OR = 1.13; 95% CI: 0.23 to 5.45, *P*=0.88; *P*=0.94 for heterogeneity) (Table 2).

As for the safety comparison between the two groups, data on postoperative complications and mortality were collected. The overall complications were reported by five studies, with 70 patients in the laparoscopic group and 88 patients in the open group [10–14]. No significant difference was found between the groups (OR = 0.43; 95% CI: 0.11 to 1.77, *P*=0.25; *P*=0.07 for heterogeneity) (Table 2). Specific complications were further analyzed, including gastrointestinal bleeding, intra-abdominal abscess, internal hernia, pneumonia, and ARDS. As is shown in Table 2, no significant difference was found in these items between the two groups (all *P* > 0.05). As for mortality, there were 411 patients involved, with 288 patients in the laparoscopic group and 123 patients in the open group [10, 13, 15]. There was no significant difference found between the two groups regarding the mortality rate (OR = 0.56; 95% CI: 0.15 to 2.12, *P*=0.40; *P*=0.45 for heterogeneity) (Table 2).

### 3.4. Sensitivity Analysis

As significant heterogeneity existed, sensitivity analysis was conducted in the following items: operating time, overall complications, and gastrointestinal bleeding. As for operating time, heterogeneity remained high while carrying out the sensitivity analysis. The pooled results changed into no significant difference between the two groups after the removal of Redwan et al.'s study (OR = −18.31; 95% CI: −39.33 to 2.72, *P*=0.09; *P*==0.0003, *I*2 = 84% for heterogeneity) [14], Oida et al.'s study (OR = −37.57; 95% CI: −82.60 to 7.46, *P*=0.10; *P* < 0.00001, *I*2 = 94% for heterogeneity) [12], and Khaled et al.'s study (OR = −29.64; 95% CI: −61.18 to 1.89, *P*=0.07; *P* < 0.00001, *I*2 = 94% for heterogeneity) [13]. As for overall complications, the pooled result remained unchanged while carrying out the sensitivity analysis. After removal of Khaled et al.'s study [13], the heterogeneity disappeared (OR = 0.88; 95% CI: 0.32 to 2.41, *P*=0.80; *P*=0.89, *I*2 = 0% for heterogeneity). As for gastrointestinal bleeding, the pooled results remained unchanged while carrying out the sensitivity analysis. However, the heterogeneity disappeared when Khaled et al.'s study (OR = 6.58; 95% CI: 0.97 to 44.54, *P*=0.05; *P*=0.56, *I*2 = 0% for heterogeneity) [13] or Redwan et al.'s study (OR = 1.34; 95% CI: 0.30 to 5.97, *P*=0.70; *P*=0.16, *I*2 = 45% for heterogeneity) [14] was deleted.

### 3.5. Publication Bias

**Figure 2:**
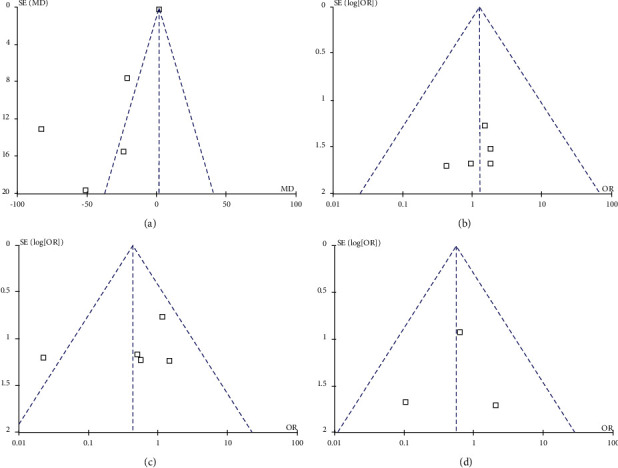
Funnel plot for results from included studies comparing the safety and efficacy of the surgical intervention for pancreatic pseudocyst between the laparoscopic approach and the open approach. (a) Funnel plot of operating time. (b) Funnel plot of successful rate. (c). Funnel plot of overall complications. (d) Funnel plot of mortality.

The funnel plots on success rate and mortality showed all the included studies lay inside the limits of the 95% CI, indicating that there was no serious publication bias (Figures 2(b) and 2(d)). As the pooled results of operating time and overall complications were not stable during the sensitivity analysis, both Begg's and Egger's tests, as well as the funnel plot, were applied to assessing the publication bias. The funnel plot on operating time showed three studies analyzed lied in the significant areas, showing an existence of publication bias (Figure 2(a)) [10, 13, 14]. Begg's tests showed a *P* value of 0.806, but Egger's tests showed a significant publication bias (*P*=0.046). The funnel plot on overall complications showed one study analyzed was in the significant areas and four studies were in the nonsignificant areas, indicating an existence of publication bias (Figure 2(c)) [13]. But, no publication bias was indicated by Begg's and Egger's tests (*P*=1.000 and *P*=0.463, for Begg's and Egger's tests, respectively). Even though both Begg's and Egger's tests on overall complications showed no publication bias, these results may be limited by the small number of the studies included.

## 4. Discussion

For complicated PPs or PPs with symptoms, aggressive surgical interventions should be performed. Apart from the traditional open surgical approach, minimally invasive surgical approaches have become increasingly preferred [16]. As few prospective studies have been conducted to compare the laparoscopic approach and the open approach interventions until today, these surgical interventions were performed mainly based on the experience of surgeons. The present study was the first systemic review to distinguish the potential differences between the two approaches.

Under the laparoscopic approach, the pseudocyst is drained to the stomach, duodenum, or the jejunum wall. The present study confirmed that the success rate of the laparoscopic approach was not inferior to that of the open approach. This result was in line with the previous studies which reported a satisfied cure rate under the laparoscopic approach [17, 18]. Moreover, the present study indicated a comparable recurrence rate between the two approaches. In the study conducted by Palanivelu et al. [19], the recurrence rate was reported as low as 1% under the laparoscopic approach, which also confirmed that the laparoscopic approach was associated with a low recurrence rate for PPs.

Considering the safety issues, the present study did not find a significant difference regarding the complications after surgical interventions between the two approaches. In the study conducted by Palanivelu et al. [19], all the surgical interventions were successfully performed, and the perioperative complications were also similar between the laparoscopic approach and the open approach. Moreover, the present study found the laparoscopic approach was not associated with increased perioperative mortality rate. In a previously published review, the mortality rate of open cystogastrostomy was reported to be 5% in contrast to 0% for the laparoscopic cystogastrostomy [20].

Other than the abovementioned items, the present study found a laparoscopic approach was associated with less intraoperative blood loss, which was similar with the result of the study conducted by Palanivelu et al. [19] The present study also showed a significant shorter operating time for the laparoscopic approach compared with the open approach. The longer operating time for the open approach may be due to the added time of opening and closing the abdomen during the surgery operation.

Because there existed some inherent limitations, consideration should be taken when interpreting the results in the present study. Firstly, the number of included studies is relatively small and none is RCT. Due to the design nature, selective bias could not be avoided. Secondly, as the included studies were conducted in different clinical centers, variations exist across the studies involving samples, surgical experiences, and clinical procedures. For example, the sizes of pseudocysts and ages were different between the laparoscopic and the open groups in the study by Melman et al. [11]. These variations might contribute to the high heterogeneity in some synthesized results. Even though the random-effects model was adopted to alleviate the heterogeneity, overcoming all the potential bias was impossible. Thirdly, during the process of data collection, an indirect data acquisition method was used. Finally, although the funnel plots in the present study indicated minimal publication bias for overall complications, the risk of publication bias always existed.

With the advanced imaging systems and excellent hemostatic techniques, and better suturing skills, most PPs can be treated with the laparoscopic approach. The present study confirmed the laparoscopy approach plays an important role in managing the PPs, with comparable cure rate and long-term outcomes. It is a safe and effective surgical modality for PPs, benefiting patients with a shorter operating time and less intraoperative blood loss. Thus, the laparoscopic approach is a promising surgical option for PPs. However, better-designed comparative studies are still needed.

## Figures and Tables

**Table 1:** Baseline characteristics and demographics of patients included.

First author, year	Number		Age		Gender (male/female)		BMI (kg/m^2^)		Etiology (alcoholic/biliary/traumatic/idiopathic/other)		Size of PP		NOS score
	LG	OG	LG	OG	LG	OG	LG	OG	LG	OG	LG	OG	
Cervantes et al., 2004	10	6	42.25 ± 14.72	36.00 ± 10.39	6/4	5/1	22.5 ± 2.8	21.2 ± 2.4	4/4/0/0/2	4/1/0/0/1	NR	NR	8
Melman et al., 2013	16	22	46.50 ± 3.6	52.0 ± 3.8^*∗∗*^	10/6	10/12	29.2 ± 1.8	28.6 ± 1.5	NR	NR	10.4 ± 0.5	9.5 ± 0.8^*∗∗*^	8
Oida et al., 2011	10	18	57.4 ± 10.8	55.4 ± 13.5	0/10	2/16	NR	NR	8/0/0/2/0	11/0/1/6/0	15.5 ± 3.1	14.2 ± 1.8	7
Khaled et al., 2014	30	10	55 ± 12.25	57.75 ± 13.01	21/9	3/7	26.0 ± 12.0	28.25 ± 4.91	11/13/0/0/0	5/3/0/0/2	10.0 ± 3.25	13.5 ± 4.63	9
Redwan et al., 2017	4	32	51.8 ± 1.9	48.8 ± 2.3^*∗∗*^	2/2	19/13	27.3 ± 1.7	29.1 ± 1.3	0/1/3/0/0	0/19/8/5/0	10.1 ± 0.8	9.9 ± 1.1	9
Wang et al., 2019	248	107	NR	NR	158/90	66/41	NR	NR	NR	NR	NR	NR	6

^a^Significant difference, ^*∗*^*P* < 0.05, ^*∗∗*^*P* < 0.01. ^b^The Newcastle–Ottawa Scale (NOS) score.

**Table 2:** Summary of pooled results.

Pooled result	Statistical method	Number of studies	MD/OR	95% CI	*P* value	Heterogeneity	
						*P*	*I* ^2^ (%)
Intraoperative blood loss	Fixed	2	−69.97	−95.14, −44.79	<0.00001^*∗∗*^	0.86	0
Operating time	Random	5	−33.12	−62.24, −4.00	0.03^*∗*^	<0.00001^*∗∗*^	93
Success rate	Fixed	5	1.25	0.33, 4.81	0.74	0.97	0
Recurrence rate	Fixed	4	1.13	0.23, 5.45	0.88	0.94	0
Overall complications	Random	5	0.43	0.11, 1.77	0.25	0.07	53
Gastrointestinal bleeding	Fixed	4	2.12	0.56, 8.02	0.27	0.11	51
Intra-abdominal abscess	Fixed	3	0.85	0.37, 1.98	0.71	0.70	0
Internal hernia	Fixed	3	0.17	0.03, 1.17	0.07	0.93	0
Pneumonia	Fixed	4	0.48	0.19, 1.20	0.11	0.91	0
ARDS	Fixed	4	0.98	0.48, 2.01	0.96	0.48	0
Mortality	Fixed	3	0.56	0.15, 2.12	0.40	0.45	0

MD, mean difference; OR, odds ratio; CI, confidence interval. ^*∗*^Statistical difference, *P* < 0.05. ^*∗∗*^Statistical difference, *P* < 0.01.

## Data Availability

The data supporting this meta-analysis are from previously reported studies and datasets, which have been cited. All data generated or analyzed during this study are included in this published article. The processed data are available from the corresponding author upon reasonable request.

## Supplementary Materials

Supplementary Materials Supplementary Table 1: the PRISMA Checklist of the present study. .
